# Reviews of Books

**Published:** 1921-12

**Authors:** 


### REVIEWS OF BOOKS.

**THE HEALTH OF THE INDUSTRIAL WORKER.** By E. L. COLLIS, M.A., M.D., and M. GREENWOOD, M.R.C.P., M.R.C.S. (J. and A. Churchill. Price 30s. net.)

" Non est vivere, sed valere, vita "—The aim of preventive medicine is not only to do away with the more serious diseases, and to postpone death, but also to enable each one to be free from even minor disabilities, to be so vigorous and strong as to reach the maximum efficiency during life, to obtain (as it is expressed, somewhat colloquially) the most out of life, and not to drag, maimed by disease or accident, through an existence miserable and wretched to the individual, costly and expensive to the State. Within the last three or four decades the advances of public health work have perhaps been mainly in the direction of obtaining better general sanitation, of gradually overcoming the numerous defects in houses and dwellings, and of overwhelming the forces of many of the infectious diseases, so that, with the exception of occupational illnesses, very little attention, until recently, has been devoted to the important subject of the general hygiene of industry. Consequently, the publication of the brilliant work of Professor Collis and Dr. Major Greenwood, on " The Health of the Industrial Worker," is particularly welcome and opportune, opening, as it does, an almost undeveloped branch of medical science.

Dealing with the historical aspect, the book reviews the evolution of industry, the development of factory and workshop, and the effect of this on disease and on national physique; and following this is an outline of the legislation which was effected to meet the difficulties encountered. Some observations are given on the principles of statistical science, and the authors advance, very justly we think, the plea that the medical student should be taught more fully and with greater lucidity the value of statistics and the methods used in dealing with them.

The essential need at the present time all over the business world is increased production, and many methods close at hand and easy to apply of effecting this are overlooked and little utilised. " The idea that if a worker can make 100 articles in one hour he ought to make 1,000 in ten hours . . . is seen to be as foolish as to expect a runner who can sprint 100 yards in 10 seconds to cover a mile in 176 seconds "; and surely this apt illustration shows the folly of expecting a large production with excessive spells of work, and demonstrates the need of regulating hours of labour and avoiding the evil results of fatigue. In the subject of ventilation is embodied all the recent work of Leonard Hill, which has exploded the old theory of $CO_2$ excess being the chief factor to watch; the introduction of the Kata thermometer now causes attention to be paid to air-movement and rate of cooling, so that the desiderata to be sought are epitomised: " Cool rather than hot—dry rather than damp—diverse in temperature in different parts and at different times, rather than uniform and monotonous, moving rather than still."

Closely connected with the problem of a greater production is the subject of the employment of women in industry, " as no nation can afford to leave any source of energy unused, and certainly not half its population, unless an overpowering racial reason exists." Dealing with the problem in a broad manner, the conclusion drawn seems to point to the fact that certain characteristics of women are of definite value in the evolution of industry (which it is claimed was primarily developed by them), and that their employment bears a beneficial influence by reason of the effect on legislation. It must not be forgotten, however, that there is yet a lack of accurate information on the effect of occupation on women.

Perhaps one of the best sections of the book is that on the epidemiology of tuberculosis. Concerning the effect of industrial life on the incidence of this disease, the recent war acted as a vast experiment of Nature owing to the tremendous increase in the numbers of women employed, and the data derived from this seems to point to the conclusion that factory life, definitely though indirectly, causes an increase in tuberculosis by sensitising the worker to react more sharply to the unwholesome home conditions. It is well that we should in this way be reminded of the importance of the *soil* (the individual) on which the *seed* (the bacillus) falls in the production of disease, for the rapid advance of bacteriology has perhaps brought the latter factor into such prominence that we are apt to forget that the conditions (the susceptibilities and idiosyncrasies) of the soil play an important rôle in deciding whether the seed perishes or " brings forth fruit, some thirtyfold, some sixtyfold, some an hundredfold."

The wealth of material contained in this book is enormous, as embodied in the various chapters is the vast extent of knowledge which has been published both in official works and in original papers. A great deal of this treats with the results derived from observations on factory life during the war, and if all this had not been collated, or if the valuable conclusions deduced had not been brought before medical men in both administrative and clinical work, not only would a unique opportunity have been lost, but the great advance which this volume will effect in the evolution of factory hygiene would have been forfeited. The amount of knowledge is only equalled by the masterly style in which the book is written, and each section has been treated in such a thorough and well-balanced way that the whole is to the reader interesting, delightful, and attractive.

The volume achieves much in that it conclusively demonstrates the tremendous influence of factory and workshop on the health of those employed, and if the means, which are both reasonable and available, for improving industrial life are utilised to the full the result will be beneficial to the worker as well as profitable to the employer. It will

**Reviews of Books—(*cont.*).**

undoubtedly bring us nearer the ideal when men will understand that, as Tolstoy wrote, "Work, the inevitable condition of human life, is the true source of human welfare," and even the factory hand may realise that "labour is the joyful business of life."—J. V. A. S.

**BURDETT'S HOSPITALS AND CHARITIES, 1921.** The Year Book of Philanthropy and Hospital Annual. 1026 pp. (The Scientific Press, Ltd., 28 and 29 Southampton Street, Strand, W.C. 2. Price 17s. 6d. net, 18s. 6d. post free.)

Were it possible to argue that "Burdett's Annual" has a rival, the admission would inevitably follow that, at present, the competitor excels this Annual only in expedition of production at the sacrifice of a quality which remains the exclusive possession of "Burdett." This well-known publication aims at and achieves the presentation of essential statistics and information regarding the charitable institutions of the British Isles for a given year. Necessarily such a compilation must be based upon the published reports of these institutions, some of which are issued with creditable promptitude, and some so long after the period to which they relate as to lose a great part of their value. But in these days of changing conditions and striking variations in cost the principle adopted for the purposes of deductions of any value cannot safely be abrogated. Better to have reliable and carefully tabulated information of the position, requirements, and finance of the hospitals and kindred charities for a given year than hastily prepared data of work in different years.

But the charities and institutions detailed in "Burdett's Hospitals and Charities," now in its thirty-second year, are not merely those belonging to our country; they include the hospitals, nursing institutions, and mental hospitals and asylums of the Dominions and Colonies and the United States of America, thus affording a world-wide view of public works of care and cure on behalf of the sick, afflicted, and needy. Of the many institutions to be found in the directory pages, which make up the main part of the work, a large number afford the material upon which is based the summarised information and editorial comment contained in the preliminary chapters. Here we find, prepared, surely, at a cost of time and trouble that cannot easily be realised, tables showing the volume of income and expenditure of 1,871 charities, including administrative and collecting societies, in the British Isles in the year 1919. The year is an interesting one, as, the Armistice past, the deceptive prosperity of institutions and individuals under war conditions was on the wane. This change is, briefly, although possibly insufficiently, exemplified by the fact that while in 1918 the institutions had a surplus of income over expenditure of £1,721,623 (capital gifts and extraordinary expenditure included), the surplus had fallen in 1919 to £968,381. Proportionately, the declining fortunes of the voluntary hospitals are even more striking, the expenditure for 1919 being but £55,145 above the income, after the substantial surplus of three-quarters of a million in 1918.

Following upon the chapter quoted are interesting summaries of the work of King Edward's Hospital Fund, the League of Mercy, Hospital Sunday and Saturday Funds, and chapters on hospital finance. The excellent index runs to over sixty pages. A general "tightening-up" process has been adopted, and institutions closed for more than two years are omitted, whilst particulars are curtailed of institutions that are so blind to their interests as not to furnish reports. No doubt this policy will lead to the earlier publication of the Annual, and on this point, as on the general excellence of the work, the Editor and his assistants must be cordially congratulated.—G. H. H.

**THE STAGES OF HUMAN LIFE.** By J. LIONEL TAYLER, M.R.C.S. Pp. xiv, 377. With illustrations. (London: John Murray. 1921. Price 18s. net.)

In this volume the author has set himself the task of attempting to present the principal facts connected with the mental and physical development of the human body in such a manner as to create interest in and to inspire a reverence for its life, when duly controlled by sane desires and wise motives. He writes for the general reader, on the whole, for whose benefit a glossary of terms used appears, but it is not implied thereby that it would be uninteresting to medical men. On the contrary, many of its chapters will provide much food for thought and at the same time may furnish useful material for addresses or home talks to patients. The characteristic tendencies belonging to each age-period are discussed from the health standpoint, and the paths that lead to the fullest realisation of life's possibilities are indicated in connection with each. The preservation throughout life of the aroma that is peculiar to adolescence is regarded as the key to happiness, for if we do not dissipate this gift, it reveals to us the infinity and the beauty of the works of nature, while "a debasement of our powers, a wrong development of our powers, a rationalisation of our feelings, causes us to see in the shadow of mechanism a spiritual death." We can forgive a certain discursiveness of style in the recognition of the author's earnest plea for the necessity of a naturalistic outlook upon life which should be directed throughout by healthy desires alone. Parents and teachers, as well as medical men, will welcome the many helpful suggestions contained in this book.—G. N. M.

**BOTANY FOR STUDENTS OF MEDICINE AND PHARMACY.** By F. E. FRITCH, D.Sc., Ph.D., and E. J. SALISBURY, D.Sc. (London: G. Bell and Sons, Ltd. Price 10s. 6d. net.)

The authors are to be congratulated on having compiled an excellent and well-illustrated handbook in which the elements of botany are clearly presented. They have embodied in their book the results of their considerable experience in the teaching and examination of medical students; and this volume appears adequately to cover the field of knowledge in which the examination candidate is

**Reviews of Books—(*cont.*).**

expected to be familiar. Professor Boycott, the Gresham Professor of Pathology in the University of London, has written a preface of which we quote the conclusion: "The business of practical medicine is to make people feel better, and it does this by helping on the natural processes of repair and restoration which are common to man and the earthworm; the mending of a broken leg depends on what the body does, the mending of a broken bicycle on what the engineer does. If anyone wants to play a good doctor's part . . . he ought to know biology, he wants to read his patients in the original. Besides, it makes medicine so much more amusing."—A. B.

**MATERIA MEDICA, PHARMACY, PHARMACOLOGY AND THERAPEUTICS.** By W. HALE-WHITE, K.B.E., M.D. Seventeenth Edition. (London: J. and H. Churchill. Price 1[illegible]s. 6d. net.)

Some lucky people have brains which seem to be the storehouses of figures. They can remember "dates," except sometimes that of the year in which they were born; they can rattle off the calorific value of foodstuffs; they know all sorts of interesting facts about record scores in various athletic fields, and they can tell you, without consulting the book, the doses and strengths of all the drugs and preparations in the British Pharmacopœia. Some students are like that, and, of course, they have no trouble in defeating their examiners. For our part, we were more modest in memory but none the less successful at the examination; for we evolved the theory that, provided we were cautious and did not order lethal doses of dangerous drugs in our prescriptions, it would not matter so very much if we were ignorant of the accurate doses of the less dangerous preparations; and apparently our examiners agreed with us in that. In practice, however, it is a very different matter; and the practitioner must know his pharmacopœia much more thoroughly than when he was a student; but he has this advantage as a practising doctor, that he can consult the book. The most-read work in our medical library is Hale-White's "Materia Medica," of which no less than seventeen editions have been published during the last twenty years. It is an admirable little volume, and with advancing years it has lost none of its authority, and still remains one of the most popular works on this all-important subject.—E. D.

**THE GATEWAY OF HEALTH.** By CHARLES E. HECHT, M.A., M.O.A. (St. Catherine Press, Stamford Street, S.E. 1. 1921. Price 12s. 6d.

We have received from the Food Education Society a copy of "The Gateway of Health," dealing with the Prevention of Diseases of the Teeth, edited by Charles E. Hecht, M.A., M.O.A. It is a compilation of various papers, addresses, reports of meetings, letters, and other communications of unequal value, and it would gain by revision and compression. It contains, however, matter of interest to the nurse and the health visitor, as well as to heads of institutions charged with responsibility for maintaining good health among large numbers of residents. A case is made out against over-consumption of sugar, jam, and "sticky" kinds of food as tending to promote dental caries, and evidence is adduced in favour of rejecting sweets and eating hard, coarse foods, apples, crusty, well-baked bread, and rusks. The writer is of opinion that too much milk is drunk both by children after infancy and by adults, and quotes a leading London dentist to the effect that "we dentists know a milk regimen is disastrous to the teeth." He maintains that after nine months not more than half a pint of milk a day is advisable if the child is to thrive. He has much to say on the need for fats and uncooked fruit and vegetables whereby a due supply of vitamines can be assured. Almost every canon of this volume is ignored in the ordinary institution dietary, and, whatever may be thought of the principles it advocates, readers will admit that it exposes the fallacy of many venerable dietetic precepts.

**THE CHARTERED SOCIETY OF MASSAGE AND MEDICAL GYMNASTICS: REGISTER OF MEMBERS.** 1920-21. (157 Great Portland Street, W. 1. Price 3s. 6d.)

The first Register of the Chartered Society of Massage and Medical Gymnastics has just been published. This Society came into existence on June 9, 1920, by the granting of a Royal Charter to the Incorporated Society of Trained Masseuses in amalgamation with the Institute of Massage and Remedial Gymnastics (Manchester), and the Register of Masseuses and Masseurs now issued is dated July 1, 1920, to July 15, 1921. The Register is clearly printed and arranged and gives the registered number, name, address, and additional qualifications in medical electricity or gymnastics of each member. The inclusion of a Place Index, giving the names of registered members classified under places, or in the case of London under districts, is particularly to be commended. It includes such distant countries as China, British East Africa, and South America. It would be an improvement were a table of contents added to future editions. The preface includes the objects of the Society, the names of the first Council and of that for 1921-22, together with a brief reference to the organisation. The privileges of registered members are set out, the rules for masseuses and masseurs and the conditions of registration which will apply during a period of grace yet to be decided on. We welcome the issue of this business-like production.

**A MANUAL OF PHYSICS.** By G. A. CROWTHER, Sc.D. Oxford Medical Publications. (London: Henry Frowde and Hodder & Stoughton. Second edition. Price 16s. net.)

The first-year medical student, and later the qualified doctor who wishes to take a Diploma in Public Health, has to be acquainted with certain principles of the science of physics. The knowledge required of these candidates by their examiners is not vast, and might perhaps be increased with advantage when we consider the importance of the subject to the science of medicine

**Reviews of Books—(*cont.*).**

in its various branches. There are many elementary and advanced text-books on the subject of physics—some, as Dr. Crowther points out, are so advanced as to confuse the elementary student, while others are written in the "kindergarten" style, and are of little value even to the matriculation candidate. This book by Dr. Crowther strikes a happy mean between these two extremes; it is satisfactory from a scientific aspect without being too complicated, and is simple without being puerile. We do not think that either the Public Health Diploma candidate or the elementary student will be required to be familiar with the work of Einstein, and we welcome, therefore, the simple and homely statement that light travels in straight lines. The first part of the book deals with mechanics and the property of matter; the second part with the subject of heat; and later sections with light, magnetism, and electricity. These sections should tell and teach the student of medicine all that he needs to know. There is an excellent collection of examination questions throughout the book relating to the various sections, and these should add considerably to the value of the book for the student. This work is well printed in clear type and contains many excellent illustrations and diagrams.—E. D.

**HEART DISEASE AND PREGNANCY.** By Sir James Mackenzie, F.R.S. (Oxford Medical Publications. Price 8s. 6d. net.)

Sir James Mackenzie must have been a trying child; his thirst for knowledge of the "how" and the "why" can have been equalled only by his caution in accepting the answer offered to him. This thirst for knowledge is still unquenched, and his caution is as strong as ever. If, in his opinion, the commonly accepted explanation of a phenomenon is unsatisfactory, he does not hesitate to say so in plain language; his worship is for the Truth as he sees it, and he has no veneration to offer to lesser gods. As he remarks, "where there is mystery there is fear," and in this book he has attempted with a large measure of success to raise the evil of mystery that enshrouds the subject of the effect of pregnancy upon the heart. The author discusses first the changes in the heart and circulation of a healthy woman during pregnancy, and calls attention to a common error in regarding the mechanical displacement of the apex-beat by the upward thrust from the uterus as an evidence of hypertrophy of the heart. Heart-failure is discussed at some length; separate chapters are devoted to Mitral Regurgitation, Stenosis, Aortic Disease, Irregularities of Rhythm, Auricular Flutter, and a short account is given of the neurotic heart, with some useful hints upon the inter-relationship of sexual experiences and heart-symptoms. The significance of murmurs is considered; the author's views in this matter are almost universally accepted, though it is still too true that "hasty conclusions formed by the early observers in regard to the significance of murmurs hang like a millstone round the necks of the profession." Modern views of cardiology are clearly sketched without too much detail, and the different symptoms and signs are discussed with authority. The author's conclusions are the outcome of long and continuous study of the question; his patients have been watched before, during, and after pregnancy, and in the light of experience so obtained certain definite data are offered to the profession. In this little book the inquirer will find reliable answers to such questions as: Should this woman be allowed to become pregnant? Should she be allowed to have more than one child? If the pregnancy is causing distress how can she be helped, and when is it advisable to terminate the pregnancy prematurely? The teaching is dogmatic, but the dogmatism is that of a man who having first realised his own ignorance has set himself successfully to become the master of his subject.—C. E. S.

**MANUAL OF PHYSIOLOGY,** for Students and Practitioners. By Lyle and de Souza. Second edition. 824 pages. Price 21s. (Oxford Medical Publications: Henry Frowde and Hodder & Stoughton. "Lancet" Building, London, 1921.)

That a sound knowledge of physiology is the basis of medicine is well known to all of us—we must know normal conditions before we can appreciate what is abnormal. This second edition of Dr. Lyle's manual of physiology is welcome, and in it all the essential facts of physiology are presented clearly and carefully. There are over 130 excellent diagrams and illustrations, and this work, in common with others from the Oxford Medical Publications, is very well produced and admirably printed. We can recommend this manual to all practitioners who wish to be up to date in the growing science of physiology; and consider that the book will be of the greatest value to medical students who are faced with an imminent examination, in which they are naturally expected to show a knowledge of the recent advances in physiology.—A. B.

**MEDICINE FOR NURSES.** By John Henderson, M.D., Ch.B., F.R., F.P.S.G. (London: Edward Arnold. Price 8s. 6d. net.)

This book is intended to supply a need arising from the advances of recent years in the education of nurses. The author's long experience as lecturer and examiner in medical nursing in the Glasgow Royal Infirmary, and his association with nurses in actual clinical work, should entitle him to speak with authority upon the subject. Obedience and loyalty, essential qualities in a nurse, are of little use in the absence of intelligent co-operation in the treatment of disease. The author has condensed in a small volume an outline of the causes, symptoms, and treatment of disease, and has discussed in general terms the underlying principles which guide such treatment, a feature which adds much to the value of the work. The clear introductory chapters should be as helpful to the matron or tutor responsible for the training of nurses as to the nurse who seeks to be efficient in the practice of her profession. The chapter dealing with personal attendance upon the sick is rich in information of much practical value. We are glad to note that the subjects of medicines, their administration and action, and poisoning and its treatment have been dealt with at some length, for these are subjects upon which, unfortunately, many nurses are not well informed.

